# Factors associated with the ownership and use of insecticide-treated nets in Guinea: an analysis of the 2018 Demographic and Health Survey

**DOI:** 10.1186/s12936-023-04463-z

**Published:** 2023-01-26

**Authors:** Ousmane Oumou Diallo, Ifeoma D. Ozodiegwu, Alioune Camara, Beatriz Galatas, Jaline Gerardin

**Affiliations:** 1 Department of Preventive Medicine and Institute for Global Health, Northwestern University, Chicago, USA; 2 National Malaria Control Programme, Conakry, Guinea; 3 Global Malaria Programme, World Health Organization, Geneva, Switzerland

## Abstract

**Background:**

Malaria is a leading cause of outpatient visits and deaths among children in Guinea. Despite several mass distribution campaigns of insecticide-treated nets (ITNs) in Guinea, ITN ownership and use remain low. Identifying the underlying factors affecting household ITN ownership and ITN usage among those with access will allow the Guinea National Malaria Control Programme to develop targeted initiatives to improve bed net ownership and usage.

**Methods:**

To understand national and regional drivers of ITN ownership and use, multivariable binary logistic regression models were applied to data from the 2018 Demographic and Health Survey to identify risk factors of household ITN ownership and risk factors of ITN use among individuals with access. Akaike Information Criterion (AIC) was used for model parameter selection. Odds ratios were estimated with corresponding 95% confidence intervals.

**Results:**

The proportion of households in Guinea with at least one ITN was 44%, ranging from a low of 25% in Conakry to a high of 54% in Labé. Use of ITNs among those with access was 66.1% nationally, ranging from 35.2% in Labé to 89.7% in N'zérékoré. Risk factors for household ITN ownership were household size, marital status of the household head, education level of the household head, and region. For ITN use among those with access, risk factors were age, wealth quintile, marital status, and region. In the seven regions of Guinea and capital of Conakry, risk factors for household ITN ownership were household size in Boké, Faranah, and Kankan; education level of the household head in Boké, Faranah, and N’zérékoré; age of the household head in Conakry and Labé; children under five in the household in Kankan; and wealth quintile in Mamou. For ITN use among those with access, risk factors were marital status in Conakry, Faranah, Kindia, Labé, Mamou, and N’zérékoré; place of residence in Labé; children under five in the household in Labé; wealth quintile in Mamou; and age in Faranah and N’zérékoré.

**Conclusions:**

This analysis identified national and region-specific factors that affect ownership and use among those with access in Guinea. Future ITN and social-behavioural change campaigns in Guinea may particularly want to target larger households, households without children, and areas with lower perceived risk of malaria if universal coverage and usage are to be achieved for optimal malaria prevention.

**Supplementary Information:**

The online version contains supplementary material available at 10.1186/s12936-023-04463-z.

## Background

Insecticide treated-nets (ITNs) are amongst the most effective vector control tools in malaria-endemic countries, and an estimated 68% of the decline in malaria parasite prevalence in Africa between 2001 and 2015 has been attributed to ITNs [1, 2]. The use of ITNs in children under five years of age in endemic areas significantly reduces malaria episodes, severe illness, and malaria-related deaths [3]. When used at high coverage, ITNs reduce the longevity of *Anopheles* mosquitoes, and thereby the vectorial capacity and entomological inoculation rate, and thus provide both individual and community protection in endemic areas [4].

**Fig. 1:**
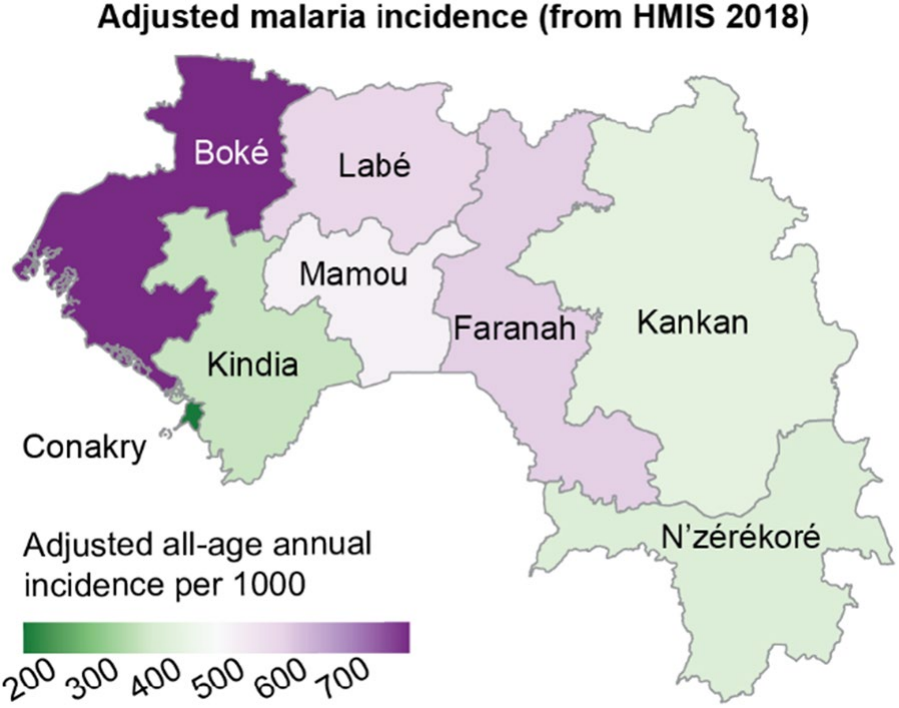
Malaria incidence in 2018 from routine case reporting via HMIS, adjusted for testing rate, reporting rate, and treatment-seeking

Guinea is a malaria-endemic country with seasonal and heterogeneous transmission [5] (Fig. 1), and malaria remains a leading cause of death among children [6]. Beginning in 2009, Guinea’s National Malaria Control Programme (NMCP) implemented ITN mass distribution campaigns every three years as a malaria prevention strategy [7]. The 2016 ITN campaign preceding the 2018 Demographic and Health Survey (DHS) distributed long-lasting insecticidal nets (LLINs) and was financed by several donors including the Global Fund to Fight AIDS, Tuberculosis, and Malaria, the United States President’s Malaria Initiative, the Senegal River Basin Development Organization (OMVS), and the World Bank [8]. All the ITN distribution campaigns in Guinea aimed to increase minimal coverage (households with at least one ITN), achieve universal coverage at the household level (all households at risk have at least one ITN for every two people), achieve universal coverage at the population level (all population at risk lives in a household with at least one ITN for every two people), and increase ITN use [9]. The ITN campaigns ultimately sought to increase the personal protection of individuals when sleeping at night [7]. Identifying factors underpinning gaps in net access and gaps between net access and use is key to inform future ITN campaigns in Guinea.

ITN use has been shown to correlate with socioeconomic and intrinsic factors, to vary seasonally, and to vary at a subnational level. Studies conducted in some countries suggest a positive association with rural residence, educational attainment, household size, and good net quality [10, 13]. In countries of West Africa, ITN usage in the dry season tends to be lower due to high levels of heat and humidity exacerbated by lying under a net [14, 15]. Regional variation in climate, environment, cultural practice, and perceived risk may further modulate net use at the subnational level. Many high-burden countries are increasingly developing subnational intervention strategies, which often include sub nationally-tailored approaches to vector control according to specific targeting criteria [16]. Understanding the drivers of regional variation in ITN use could allow Guinea’s NMCP to develop region-specific strategies to increase ITN use and thus the impact and cost-effectiveness of vector control.

To help maximize the potential impact of ITNs in future mass distribution campaigns, this study aimed to understand the level of ITN ownership, access, and use in Guinea, and to identify the underlying factors that affect ownership and use among those who have access, at the national and regional levels.

## Methods

### Data source and variable definitions

Guinea’s 2018 Demographic and Health Survey (DHS) was used for this study [17]. The DHS collected information from a nationally and sub-nationally representative sample of Guinean households using a cluster sampling methodology. Clusters were sampled from urban and rural areas in the seven Guinean regions and from the capital of Conakry, which has no rural areas.

The household database available from the DHS Program was used for the analysis of household ITN ownership and sufficiency household ITNs. Household ITN ownership was defined as the presence of at least one ITN in the household, and household ITN sufficiency was defined as the household owning at least one ITN for every two household members; this is the same definition as universal coverage at the household level. Household ITN sufficiency can provide a misleading picture of the success of an ITN distribution programme [8] and thus is considered in this study only as a secondary indicator and not used in the later multivariable logistic regression analysis.

The household member database was used for the analysis of individual-level all-age access and ITN use among those who have access. At the individual level, ITN access was defined as the proportion of the *de-facto* population that could have slept under an ITN, assuming two persons per net [18]. The access variable was corrected to 100% when the number of ITNs per two household members was greater than one. ITN use was defined as the proportion of *de-facto* members of a household who slept under a treated net the night before the survey in households where there was at least one ITN for every two persons.

Descriptions of all variables included in the analysis are shown in Table 1. Initial selection of covariates was based on a literature review and expert opinion [11–13, 19].

**Table 1:** Variables included in the study

Variables	Level	Description
Household ITN ownership	Household	Proportion of households with at least one ITN (0 = no, 1 = yes)
Household ITN sufficiency (universal coverage at the household level)	Household	Proportion of household owning at least 1 ITN for 2 people (0 = no, 1 = yes)
Access to ITN	Individual	Proportion of the population with access to an ITN within the households
ITN use among those who have access	Individual	Person slept under the ITN the night before the survey (0 = no, 1 = yes)
Age of household head	Household	Age of household head in years
Age of individual	Individual	Age of individual in years
Sex of household head	Household	Sex of household head
Sex of individual	Individual	Sex of individual
Educational attainment	Household and Individual	Level of education of the household head or of the individual
Household size	Household and Individual	Number of de facto members
Number of rooms	Household	Number of rooms in the household
Wealth index	Household	Composite measure of household living standard calculated using data on household ownership of selected assets, housing quality, water, and sanitation facilities. The wealth index is available from the DHS survey datasets
Marital status	Household and Individual	Marital status of the household head or of the individual
Children under five	Household	Presence of children under five in the household
Pregnant status	Individual	Presence of 1 or more pregnant women in the household, or individual pregnancy status
Place of residence	Household and Individual	Type of place where the household or individual resides (urban/rural)
Region	Household and Individual	Region in which the household or individual resides

### Statistical analysis

All analysis was performed in R (version 4.2.1) and the analysis code is publicly available (see Availability of data and materials). The svydesign function (survey R package v4.0) was used to account for the complex sampling design of DHS surveys. The following steps were performed for each analysis: (A) assessment of risk factors for household ITN ownership, analysed at the household level, and (B) assessment of risk factors for ITN use among those with access, analysed at the individual level. First, associations between dependent variables and independent variables were evaluated in a bivariate analysis with the Rao-Scott Chi-square test of independence. Next, risk factors were analysed with multivariable regression models using the survey R package (v4.0) nationally and separately for each region. Odds ratios were estimated for each variable with corresponding 95% confidence intervals (CIs) and p values (alpha level = 0.05). The variables to be included in the multivariate model were selected based on the value of the Akaike Information Criterion (AIC) [20] in order to optimize model performance via the function STEPAIC from the MASS R package (v7.3–53.1). Model selection did not take into account the significance in the bivariate analysis or the univariate model.

## Results

### ITN ownership, access, and use in Guinea

**Fig. 2:**
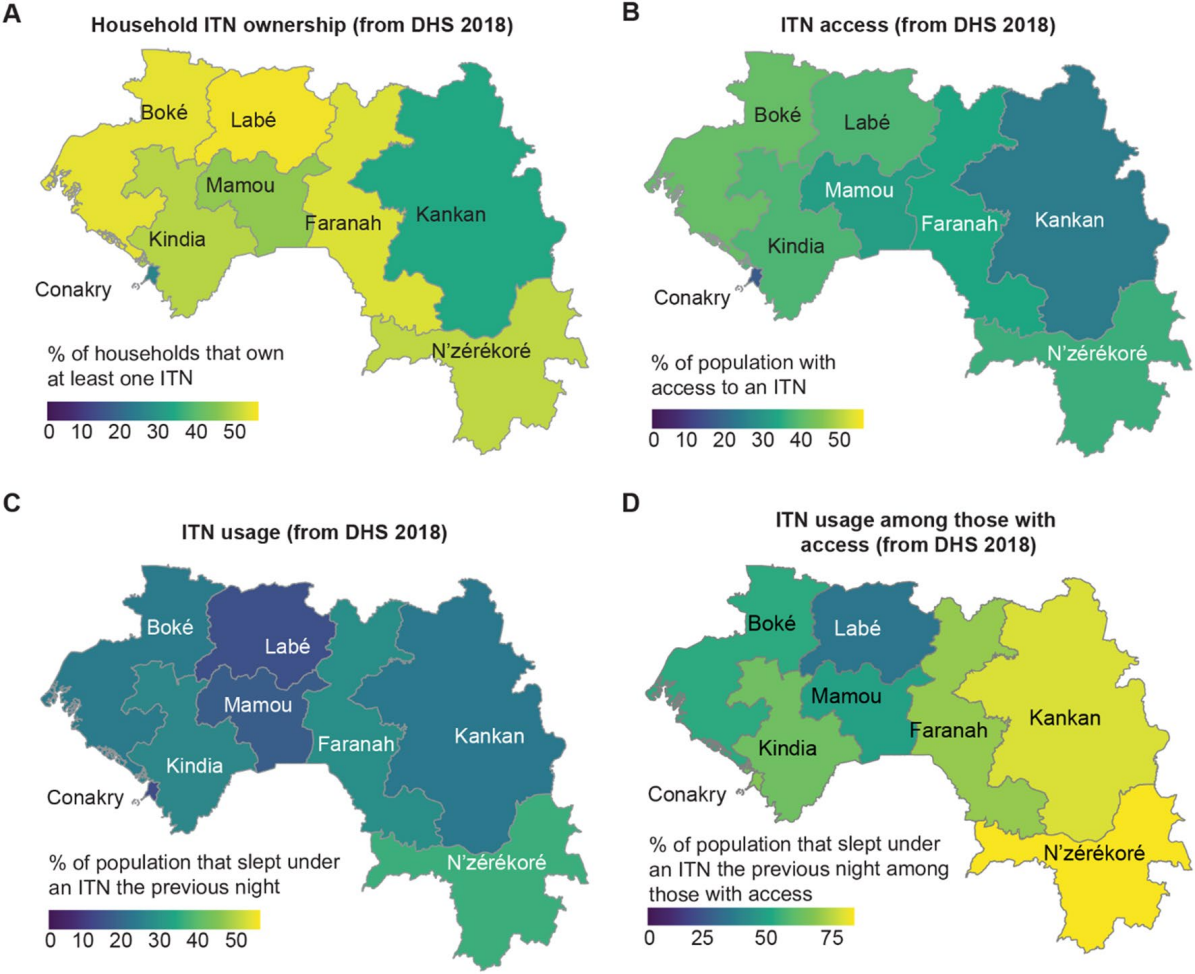
Regional variation in Guinea of **A** household ITN ownership; **B** proportion of the population with access to an ITN; **C** ITN usage; and **D** ITN usage among those with access

**Fig. 3:**
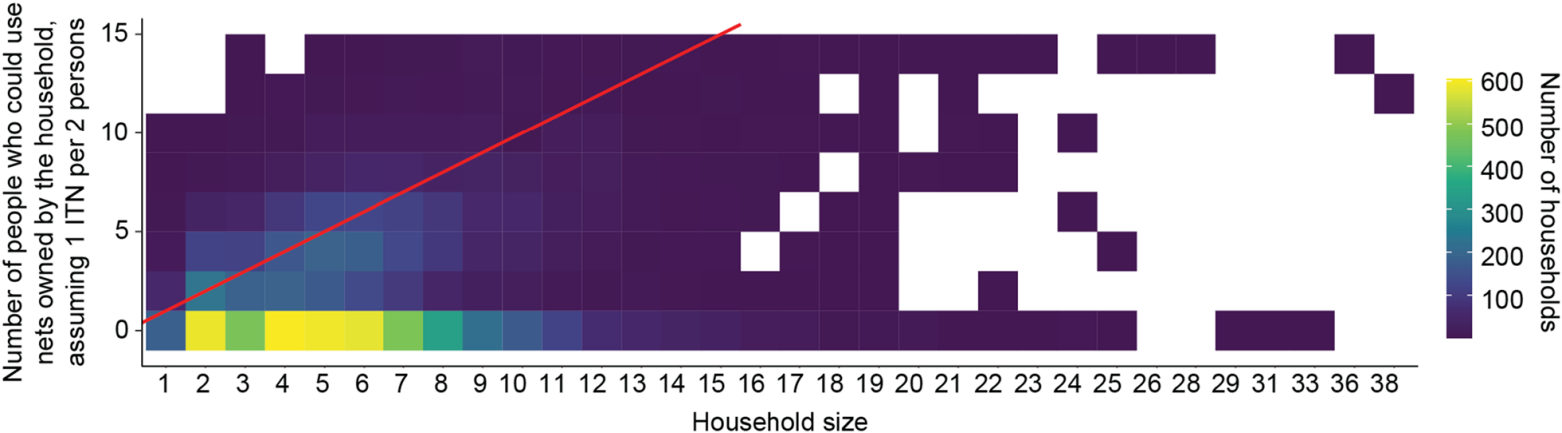
Two-dimensional histogram showing the number people who could use nets owned by the household if all nets were in use, stratified by household size, in the 2018 Guinea DHS. Yellower squares indicate combinations of number of people potentially coverable and household sizes that were more commonly observed in the DHS, darker squares indicate less-observed combinations, and white areas indicate combinations that did not appear in the DHS. The red line indicates a perfect allocation, where the number of ITNs owned by the household is exactly sufficient to cover all household members. Households above and to the left of the line have more ITNs than household members need (over-allocated with ITNs) and households under and to the right of the line have fewer ITNs than needed to cover all household member (under-allocated with ITNs)

First, the coverage of the main indicators in Guinea found in this study is as follows: the proportion of households with at least one ITN at the national level was 44% (Fig. 2A). This proportion was highest in Labé (54%) and lowest in Conakry (25%) (see Table 2 for values for all regions). The proportion of households with universal ITN coverage (at least one ITN per two persons, also referred to as ITN sufficiency) was 16.9%, ranging from 7.8% in Conakry to 25.3% in N’zérékoré. Fifty-six percent (56%) of Guinean households had no ITN, and an additional 27.1% of households owned an ITN but did not have enough for all members (Fig. 3). Population access to ITNs was 31% nationally and lowest in Conakry (16%) (Fig. 2B). ITN use was low nationally (22.9%) (Fig. 2C). ITN use among those with access was 66.1% nationally, ranging from 35.2% in Labé to 89.7% in N'zérékoré (Fig. 2D).

**Table 2:** Characteristics of survey participants and results of a bivariate analysis of household ITN ownership and sufficiency based on the Rao-Scott Chi-square test of independence

Explanatory variables	Number of households in survey, N (%)	Household ITN status			Bivariate analysis P value (Has at least one ITN)	Bivariate analysis P value (Has sufficient ITNs)
		No ITN, %	Has at least one ITN, %	Has sufficient ITNs, %		
Number of household members					< 0.001	< 0.001
1–4	2655 (33.6)	59.2	40.8	27.1		
5–7	3127 (39.5)	55.3	44.7	14.5		
> 7	2128 (26.9)	53.5	46.5	7.0		
Number of rooms in the household					0.4	0.2
1–3	5896 (74.5)	56.7	43.3	16.3		
4–6	1786 (22.6)	54.4	45.6	18.2		
> 7	228 (2.9)	54.8	45.2	15.9		
Sex of household head					0.3	< 0.001
Male	6430 (81.3)	55.8	44.2	15.7		
Female	1481 (18.7)	57.6	42.4	21.3		
Marital status of the household head					< 0.001	< 0.05
Married	6739 (85.5)	55.4	44.6	16.1		
Never married	178 (2.3)	73.7	26.3	17.2		
Divorced	133 (1.7)	58.3	41.7	25.7		
Widowed	827 (10.5)	56.5	43.5	21.1		
Age of household head					< 0.001	< 0.001
< 30	931 (11.8)	63.4	36.6	14.0		
30–40	1834 (23.2)	55.4	44.6	15.3		
40–50	1633 (20.7)	54.8	45.2	14.1		
50–60	1649 (20.9)	54.8	45.2	17.8		
> 60	1861 (23.5)	55.3	44.6	20.9		
Education level of the household head					0.8	0.6
None	5321 (67.3)	55.8	44.2	16.9		
Primary	767 (9.7)	55.0	45.0	17.7		
High	1780 (22.5)	57.3	42.7	15.8		
Presence of children under five					< 0.001	< 0.001
Yes	5267 (66.6)	54.6	45.4	12.2		
No	2443 (33.4)	59.2	40.8	25.7		
Wealth quintile					< 0.001	< 0.001
Lowest	1698 (21.5)	51.4	48.6	16.2		
Second	1601 (20.2)	53.2	46.8	18.6		
Middle	1534 (19.4)	51.2	48.8	21.5		
Fourth	1588 (20.1)	61.5	38.5	13.6		
Highest	1488 (18.8)	64.0	36.0	13.8		
Place of residence					< 0.001	< 0.001
Urban	2701 (34.1)	64.4	35.6	11.9		
Rural	5210 (65.9)	51.8	48.2	19.2		
Region					< 0.0001	< 0.001
Boké	801 (10.1)	47.0	53.0	22.7		
Conakry	1265 (16.0)	75.2	24.8	7.8		
Faranah	670 (8.5)	47.9	52.1	13.6		
Kankan	1090 (13.8)	66.9	33.1	8.1		
Kindia	1202 (15.2)	51.3	48.7	25.3		
Labé	895 (11.3)	45.9	54.1	24.4		
Mamou	741 (9.4)	54.5	45.5	17.3		
N’zérékoré	1245 (15.7)	50.5	49.5	17.1

### Sociodemographic factors associated with household ITN ownership and sufficiency

7910 households were included in a 2-step analysis. First, a bivariate analysis with the Rao-Scott Chi-square test of independence was used to quantify associations between household ITN ownership and individual covariates at the national level (Table 2); this was repeated with household ITN sufficiency and the same set of covariates. The bivariate analysis was an exploratory analysis to understand basic relationships between ownership or sufficiency and covariates of interest. Next, to understand the risk factors associated with ITN household ownership, a univariate logistic regression was used to identify covariates that are risk factors when taken individually; then multivariable logistic regression was used to identify covariates that remained risk factors when other variables were taken into account. The same set of independent variables was used for logistic regression as in the bivariate analysis (Table 3).

**Table 3:** Risk factors associated with household ITN ownership

Explanatory variables	Univariate analysis		Multivariable analysis	
	OR	95% CI	OR	95%CI
Number of household members				
1–4	Ref.		Ref.	
5–7	1.17^**^	1.04–1.33	1.09	0.96–1.23
> 7	1.26^*^	1.10–1.45	1.18^*^	1.02–1.37
Number of rooms in the household				
1–3	Ref.			
4–6	1.09	0.96–1.25		
> 7	1.08	0.78–1.50		
Sex of household head				
Male	Ref.			
Female	0.93	0.86–1.06		
Marital status of the household head				
Married	2.26^***^	1.55–3.30	1.7^*^	1.15–2.52
Never married	Ref.		Ref.	
Divorced	2.01^**^	1.18–3.41	1.54	0.90–2.62
Widowed	2.16^***^	1.43–3.25	1.65^**^	1.10–2.55
Age of household head				
< 30	0.70^***^	0.58–0.85	0.81^*^	0.66–1.00
30–40	0.98	0.84–1.13	1.06	0.91–1.23
40–50	Ref.		Ref.	
50–60	1.00	0.87–1.16	1.0	0.86–1.16
> 60	0.98	0.85–1.13	1.01	0.87–1.17
Education level of the household head				
None	Ref.		Ref.	
Primary	1.03	0.87–1.23	1.09	0.92–1.31
High	0.94	0.81–1.10	1.35^***^	1.17–1.56
Presence of children under five				
Yes	1.21^***^	1.09–1.35		
No	Ref.			
Wealth quintile				
Lowest	Ref.		Ref.	
Second	0.93	0.78–1.11	1.20	0.98–1.46
Middle	1.01	0.84–1.21	1.10	0.9–1.34
Fourth	0.66^***^	0.54–0.81	1.12	0.81–1.55
Highest	0.60^***^	0.48–0.74	1.00	0.68–1.49
Place of residence				
Urban	Ref.		Ref.	
Rural	1.68^***^	1.42 – 1.99	1.3	1.0–1.51
Region				
Boké	1.15	0.80–1.64	1.18	0.82–1.69
Conakry	0.34^***^	0.24–0.47	0.38^***^	0.26–0.55
Faranah	1.10	0.77–1.58	1.13	0.79–1.62
Kankan	0.50^***^	0.36–0.70	0.53^***^	0.38–0.74
Kindia	0.97	0.7–1.35	1.03	0.74–1.43
Labé	1.20	0.86–1.66	1.27	0.92–1.77
Mamou	0.85	0.6–1.21	0.89	0.62–1.26
N’zérékoré	Ref.		Ref.	

p value: * < 0.05; ** < 0.01; *** < 0.001

The bivariate analysis at national level found that the number of rooms, the sex of the head of household, and the education level of the head of household were not significantly associated with ITN ownership, whereas all remaining variables were. Larger households were more likely to own an ITN than small households, but small households were more likely to have sufficient ITNs than large households. By marital status of the household head, the highest ITN ownership was observed among households with a married household head and highest ITN sufficiency among those with a divorced head. By age of the household head, the highest ITN ownership was observed among households with a head aged > 40 years and highest ITN sufficiency among those with head aged > 60 years. Households with children under five had higher ITN ownership and sufficiency than those without children under five. By wealth, the highest ITN ownership and sufficiency were observed among households in the middle wealth quintile. Rural areas had higher ITN ownership and sufficiency than urban areas. By region, the highest ITN ownership was observed in Labé and the highest sufficiency in Kindia.

In multivariable regression models of national household ITN ownership (Table 3), large households had a significantly greater likelihood of ownership than small households (aOR = 1.18, CI = 1.02–1.37). Household ITN ownership was more likely among households with married or widowed heads compared those with never-married heads (aOR = 1.7, CI = 1.15–2.52 and aOR = 1.65, CI = 1.10–2.55, respectively). Compared to households with heads of household aged 40–50 years, those with heads aged < 30 years were significantly less likely to own at least one ITN (aOR = 0.81, CI = 0.66–1.0). Households with heads with high education levels had a greater likelihood of ITN ownership than those with no education (aOR = 1.35, CI = 1.17–1.56). Household ITN ownership was significantly higher in rural households than urban households (aOR = 1.22, CI = 1.0–1.51). Regional differences in household ITN ownership were observed. Household ITN ownership was less likely in Conakry (aOR = 0.38, CI = 0.26–0.55) and Kankan (aOR = 0.58, CI = 0.38–0.74) than N’zérékoré.

The multivariable analysis was repeated separately for each region (Additional file 1: Tables S1.1-S1.8). Large households had a significantly greater likelihood of ITN ownership than small households in Boké (aOR = 1.39, CI = 1.07–1.86), Faranah (aOR = 1.61, CI = 1.15–2.25), and Kankan (aOR = 1.65, CI = 1.10–2.45). Households with heads with high education levels had a greater likelihood of ITN ownership than those with no education in Boké (aOR = 1.44, CI = 1.03–2.01), Faranah (aOR = 1.67, CI = 1.11–2.5), and N’zérékoré (aOR = 1.53, CI = 1.01–2.31). In Conakry, compared to households with head of household aged 40–50 years, all other households excepting those with heads 30–40 years were significantly less likely to own at least one ITN; in Labé, only households with heads aged > 60 years were significantly less likely to own at least one ITN (aOR = 0.57, CI = 0.33–0.99). Households with children under five had significantly greater likelihood of ITN ownership than households without children under five in Kankan (aOR = 1.68, CI = 1.08–2.59). Households in the fourth and highest wealth quintiles had significantly greater likelihood of ITN ownership than the lowest quintile in Mamou (aOR = 1.73, CI = 1.06–2.82 and aOR = 2.98, CI = 1.49–5.96).

### Sociodemographic characteristics associated with ITN use among individuals with access

Of 48,916 total individuals included in this analysis, 16,751 individuals had access to ITNs, and their ITN usage was further analysed. The same 2-step approach was used as was done for household ITN ownership to first quantify associations between individual covariates and ITN usage given access using a bivariate analysis (Table 4), then identify risk factors associated with ITN use among individuals with access using multivariable logistic regression (Table 5).

**Table 4:** Characteristics of survey participants of ITN use among individuals with access and results of a bivariate analysis

Explanatory variables	Number of participants who slept in the household the night before the survey, N (%)	ITN use among those who have access		Bivariate analysis P value
		%	95% CI	
Number of household members				0.4
1–4	8006 (16.4)	66.8	62.9–70.7	
5–7	18,537 (37.9)	69.8	67.1–72.5	
> 7	22,372 (45.7)	68.1	64.8–71.5	
Number of rooms in the household				0.009
1–3	31,158 (63.7)	70.4	67.9–72.9	
4–6	14,854 (30.4)	64.5	60.9–68.1	
> 7	2904 (5.9)	69.6	61.6–77.5	
Sex				0.11
Male	23,066 (47.2)	65.3	65.3–70.4	
Female	25,850 (52.8)	66.8	66.8–71.5	
Education				< 0.001
None	30,120 (61.6)	69.4	66.9–71.9	
Primary	11,579 (23.7)	64.6	61.6–67.6	
High	7146 (14.6)	71.4	68.1–74.7	
Age				< 0.001
0–5	9685 (19.8)	73.2	70.6–75.7	
5–10	8639 (17.7)	62.9	59.9–65.9	
10–20	10,802 (22.1)	61.3	58.1–64.6	
20–40	10,683 (21.8)	73.7	71.1–76.3	
40–50	3284 (6.7)	72.1	68.9–75.3	
50–60	3024 (6.2)	68.6	65.1–72.1	
> 60	2798 (5.7)	67.1	63.4–70.8	
Age of household head				< 0.001
< 30	3954 (8.1)	74.9	69.7–80.1	
30–40	10,269 (20.9)	77.1	73.7–80.3	
40–50	10,909 (22.3)	69.9	66.4–73.4	
50–60	11,538 (23.6)	65.9	62.3–69.7	
> 60	12,245 (25.03)	60.1	56.4–63.8	
Marital Status				< 0.001
Married	16,375 (63.9)	73.3	71.1–75.6	
Never married	7126 (27.8)	60.2	56.3–64.2	
Divorced	449 (1.8)	69.7	61.7–77.7	
Widowed	1650 (6.4)	66.3	61.4–71.2	
Pregnancy status				< 0.001
Yes	953 (1.9)	74.8	69.3–80.3	
No	9942 (20.3)	71.5	69.0–74.1	
Presence of children under five in the household				0.002
Yes	37,037 (75.7)	69.9	67.5–72.3	
No	11,879 (24.3)	64.6	61.1–68.1	
Household wealth quintile				0.07
Lowest	9807 (20.0)	63.9	59.8–68.0	
Second	9826 (20.1)	68.2	64.1–72.2	
Middle	9834 (20.1)	69.6	65.5–73.6	
Fourth	9728 (19.9)	72.2	68.1–76.4	
Highest	9717 (19.9)	69.9	64.7–75.1	
Place of residence				0.003
Urban	31,989 (34.6)	74.1	70.1–78.1	
Rural	16,927 (65.4)	66.4	63.7–69.1	
Region				< 0.001
Boké	5069 (10.4)	55.2	48.2–62.2	
Conakry	7497 (15.3)	72.5	63.8–81.2	
Faranah	4691 (9.6)	75.2	69.9–80.4	
Kankan	7005 (14.3)	84.8	80.9–88.7	
Kindia	7195 (14.7)	67.5	60.7–74.4	
Labé	4854 (9.9)	35.2	29.3–41.1	
Mamou	4157 (8.5)	51.4	43.9–58.9	
N’zérékoré	8447 (17.3)	89.7	87.4–92.0

**Table 5:** Risk factors associated with ITN use among those who have access

Explanatory variables	Univariate analysis		Multivariable analysis	
	OR	95%CI	OR	95%CI
Number of household members				
1–4	0.94	0.77–1.16	1.15	0.91–1.45
5–7	1.08	0.91–1.29	1.13	0.93–1.37
> 7	Ref.		Ref.	
Sex				
Male	Ref.			
Female	1.06	0.99–1.15		
Numbers of rooms in the household				
1–3	Ref.		Ref.	
4–6	0.77^**^	0.65–0.90	0.88	0.72–1.1
> 7	0.96	0.66–1.40	1.01	0.66–1.54
Education level of household member				
None	0.91	0.76–1.08	0.87	0.71–1.04
Primary	0.73^**^	0.63–0.85	0.96	0.8–1.15
High	Ref.		Ref.	
Age				
0–5	Ref.		Ref.	
5–10	0.62^***^	0.56–0.69	0.77^**^	0.63–0.95
10–20	0.58^***^	0.51–0.66	0.67^***^	0.54–0.83
20–40	1.03	0.93–1.14	0.74^*^	0.57–0.96
40–50	0.95	0.83–1.09	0.78	0.57–1.1
50–60	0.8^**^	0.68–0.95	0.79	0.57–1.1
> 60	0.75^***^	0.63–0.89	0.91	0.63–1.31
Age of household head				
< 30	1.25	0.63–1.38	1.03	0.72–1.46
30–40	1.03	0.97–1.58	1.22	0.96–1.55
40–50	Ref.		Ref.	
50–60	0.68	0.55–1.52	0.97	0.78–1.22
> 60	0.98	0.55–1.28	0.83	0.65–1.05
Marital status of household member				
Married	1.81^***^	1.57–2.09	2.23^***^	1.83–2.73
Never Married	Ref.		1	
Divorced	1.33^***^	1.15–1.54	1.59^*^	0.98–2.58
Widowed	1.51^*^	1.03–2.23	1.6^***^	1.18–2.18
Pregnancy status				
Yes	Ref.		Ref.	
No	0.85	0.64–1.12	1.1	0.82–1.47
Presence of children under five in the household				
Yes	1.28	1.09–1.49		
No	Ref.			
Household wealth quintile				
Lowest	Ref.		Ref.	
Second	1.21	0.97–1.50	1.1	0.86–1.37
Middle	1.29^*^	1.02–1.64	1.22	0.95–1.56
Fourth	1.47^***^	1.13–1.91	1.49^**^	1.1–2.16
Highest	1.31	0.97–1.78	1.42	0.95–2.13
Place of residence				
Urban	Ref.		Ref.	
Rural	0.69^**^	0.54–0.88	0.79	0.56–1.12
Region				
Boké	0.14^***^	0.10–0.21	0.13^***^	0.08–0.19
Conakry	0.30^***^	0.18–0.50	0.19^***^	0.11–0.35
Faranah	0.35^***^	0.24–0.51	0.35^***^	0.23–0.85
Kankan	0.64^*^	0.43–0.95	0.56^***^	0.37–0.85
Kindia	0.24^***^	0.16–0.36	0.22^***^	0.14–0.32
Labé	0.06^***^	0.04–0.09	0.05^***^	0.04–0.08
Mamou	0.12^***^	0.08–0.18	0.11^***^	0.07–0.17
N’zérékoré	Ref.		Ref.	

p value: * < 0.05; ** < 0.01; *** < 0.001

The bivariate analysis found that household size, sex of the individual, and wealth quintile were not statistically significant, whereas all other variables were. Given access, ITN use was higher among those living in households with 1–3 rooms and lowest in those living in households with more than seven rooms. ITN use was more likely in individuals with high educational attainment. By age, the highest ITN use was observed among those aged 20–40 years and the lowest use among those aged 10–20 years. Residents of households with household head between 30 and 40 years of age had the highest usage levels. ITN use was higher among married persons than those never married, divorced, or widowed. Pregnant women were more likely to use their net than non-pregnant individuals. Individuals living in households with children under five years of age had higher ITN use than individuals living in households without a child under five. ITN use was higher among those living in urban areas than in rural areas. By region, ITN use was highest in the N’zérékoré region and lowest in Labé.

In a multivariable analysis of ITN use among those with access (Table 5), age, wealth quintile, marital status, and region remained significant. School-age children (5–10 years), adolescents (10–20 years), and younger adults (20–40 years) had lower likelihood of ITN use (aOR = 0.77, CI = 0.63–0.95; aOR = 0.67, CI = 0.54–0.83; OR = 0.74, CI = 0.57–0.96 respectively) than those aged 0–5 years. Individuals in the fourth wealth quintiles were more likely to use ITNs than individuals in the lowest wealth quintile (aOR = 1.49, CI = 1.1–2.16). Compared to persons who were never married, being married (aOR = 2.23, CI = 1.83–2.73), widowed (aOR = 1.6, CI = 1.18–2.18), and divorced (aOR = 1.59, CI = 1.0–2.58) was associated with higher likelihood of ITN use. Individuals living in N’zérékoré had higher odds of ITN use compared to all other regions.

Full results of the regional-level analyses are shown in Additional file 1: Tables S2.1-S2.8. Individuals who were married had significantly higher likelihood of ITN use than persons who never married in all regions except Boké and Kankan. By place of residence, individuals living in the rural areas had significantly lower odds of ITN use compared to those living in urban areas (aOR = 0.42, CI = 0.18–1.00) in Labé and no significant difference in all other regions. Only in Labé, individuals living in households with children under five had significantly greater odds of ITN use compared to those living in households without children under five (aOR = 1.86, CI = 1.16–3.1). By wealth quintile, only Mamou showed significant differences in ITN use, and individuals in the highest wealth quintiles were significantly more likely to use ITNs than individuals in the lowest wealth quintile (aOR = 4.93, CI = 1.86–13.1). School-age children (5–10 years) had significantly lower likelihood of ITN use than those aged 0–5 years in Faranah (aOR = 0.52, CI = 0.41–0.66) and N’zérékoré (aOR = 0.45, CI = 0.26–0.79).

## Discussion

This study analysed the 2018 Guinea DHS to identify risk factors for ITN ownership at the household level and use among individuals with access via multivariable logistic regressions. The results show that 44% of households owned ITNs, 31% of individuals had access to ITNs and of those who had access, 66.1% used them. Household ownership of ITNs allows the NMCP to know the proportion of households to which it has had access with ITN campaigns, and knowing the risk factors of this ownership is essential for targeting future ITN campaigns. This study found that in Guinea, significant risk factors for household ITN ownership were household size, marital status of the household head, education level of the household head, and region.

Household ITN ownership in Guinea was higher in large households than in small households. Small households were more likely to have sufficient access to ITNs, if they owned an ITN, but ownership rate was lower than for large households. This may be because members of large-size households are more likely to enroll in ITN mass campaigns but may not receive enough ITNs to cover all household members. In Guinea, ITN distribution policy is to distribute proportionally to household size without a cap. This distribution depends on an enumeration carried out by agents 1–2 months before the ITN distribution campaign. During this enumeration, agents give coupons to each household where a member has been found. Larger households may be more likely to have a member present at the time of distribution, which could explain larger households being more likely to own any net. Large households have a higher probability of having a child under 5 years of age or a pregnant woman in the household, which increases the likelihood of obtaining ITNs during antenatal care visits or at birth, but this mechanism appears insufficient to compensate for the lower per capita nets given to large households during campaigns [21]. The results suggest that the distribution allowance may contribute substantially to inadequate access if the implementation does not take household size sufficiently into account.

Households with married heads were more likely to own an ITN than households with unmarried heads, which could be due to children under 5 being more likely to live in households with married heads. Other studies have found mixed results: in Cameroon, unmarried heads had higher ITN ownership than married heads [22], but in Ethiopia, families with a married head of household had higher ITN ownership [23]. Households whose head had a high education level had greater household ITN ownership than households whose head had no education, similar to what has been observed in Nigeria [12].

Household ITN ownership was significantly lower in Conakry and Kankan regions compared to N’zérékoré region. In the Conakry region, low household ITN ownership could be explained by a high ratio between the high number of nets needed to provide universal coverage and the low number of locally acquired malaria incident cases [24].

Only 31% of the population has access to an ITN in Guinea, which is very low, and continuing to increase access should be a priority of the NMCP. While the use of ITNs in Guinea is ultimately more limited by access than by non-use, understanding the use of ITNs among those with access allows the NMCP to design campaigns to promote use even in the context of limited access. The results shown that ITN use was associated with age, marital status, wealth quintile, and region.

In Guinea, school-aged children (5–10 years) used ITNs less than those under 5 years of age, similar to observations in other countries [25]. These children give way to their siblings and are not likely to sleep with their parents in the same bed, unlike children under 5 [26] [27], which may lead to lower prioritization among this group if the number of ITNs in the household is insufficient [28]. Malaria prevalence in children over 5 remains high in Guinea [29], highlighting the need to improve prevention measures in this group.

Given access, individuals in the highest wealth quintile used ITNs more than those in the lowest quintiles. In other countries, ITN use among those in higher wealth quintile was sometimes found to be significantly more likely [25, 30, 31], but in other settings, persons in the lowest wealth quintile had the highest ITN use [12]. Wealthier households have more resources available (TV, radio) to listen to or watch malaria advertisements and learn about the advantages and disadvantages of using ITNs, which should have the benefit of increasing ITN use in the household [19]. However, the absence of questions on malaria knowledge in the 2018 Guinea DHS meant that it was not possible to evaluate the associations between household wealth, malaria knowledge, and ITN use.

The heterogeneous rates of use across regions even among those with access could be explained by regional variation in other factors associated with ITN use that were not examined in this study, such as malaria transmission, perception of risk, and ITN quality. The use of ITNs was highest in the N’zérékoré region, where parasite prevalence was the highest in 2012, when the most recent national prevalence survey was carried out [8]. In recent years, multiple NGOs and donors have targeted the N’zérékoré region to strengthen existing malaria prevention measures and improve the use of ITNs [32], which could also contribute to the higher use in this region. The regions with the lowest ITN use levels were Labé and Mamou, which are both areas where the perception of malaria risk by the population could be very low. Qualitative studies conducted in these areas could reveal reasons for the differences in significance of the variables.

One of the main limitations of this study is that the 2018 survey was conducted during the dry season, when ITN use is lower, and ITN use during the higher-transmission season could be somewhat higher than what was found in the survey [33]. During the dry season in West African countries, ITN use is lower due to factors such as heat and perception of lower risk. A review of the reasons for non-use of ITNs in Guinea ranked heat discomfort as one of the main factors for non-use [31].

Another limitation of this study is the lack of information about who exactly received the ITN or who has access to a household ITN if the household has insufficient ITNs. This may bias the analysis of risk factors for ITN use among those who have access. Between 2012 and 2018, household ITN ownership decreased in Guinea from 47 to 44%, but access and use increased to 25% and 31%, respectively. The number of ITNs distributed during the 2016 campaign preceding the 2018 survey was one ITN per 2 persons. The discrepancy between the number of ITNs distributed and the low access observed in the 2018 DHS is noticeable, suggesting that the retention time for nets in Guinea is less than 2 years [34]. Retention time for nets could be very heterogeneous across the country.

Future ITN distribution campaigns in Guinea can consider a region-specific distribution strategy that targets households according to the regional drivers of ITN ownership and use found in this study. Taking regional differences into account will allow the NMCP to efficiently target households that are most likely to lack access and to target individuals who do not use ITNs. While this study provides a starting point by identifying factors driving low ITN use, qualitative and operational studies are needed to understand why access and use are limited and how to improve them. Improved targeting of ITN distribution and community engagement campaigns could have a substantial impact on malaria burden and potentially allow Guinea to reach Global Technical Strategy targets for 2030.

## Conclusions

Household ITN ownership and population access to ITNs in Guinea are low, although use among those with access is high in most regions. This study finds household size, marital status of household head, and region were the factors most strongly associated with household ITN ownership and ITN use, suggesting that future ITN campaigns in Guinea may particularly want to target larger households, households without children, and areas with lower perceived risk of malaria if universal coverage and usage are to be achieved. Regional differences in ownership, access, and usage should be considered in future ITN campaigns to increase ITN usage more efficiently and provide greater coverage of ITNs in Guinea for optimal malaria prevention.

## Supplementary Information

**Additional file 1**: **Table S1.1**: Risk factors associated with household ITN ownership in Boké region. **Table S1.2**: Risk factors associated with household ITN ownership in Kindia region. **Table S1.3**: Risk factors associated with household ITN ownership in Faranah region. **Table S1.4**: Risk factors associated with household ITN ownership in Kankan region. **Table S1.5**: Risk factors associated with household ITN ownership in Labé region. **Table S1.6**: Risk factors associated with household ITN ownership in Mamou region. **Table S1.7**: Risk factors associated with household ITN ownership in N’zérékoré region. **Table S1.8**: Risk factors associated with household ITN ownership in Conakry region. **Table S2.1**: Risk factors associated with ITN use among those with access in **Labé** region. **Table S2.2**: Risk factors associated with ITN use among those with access in Mamou region. **Table S2.3**: Risk factors associated with ITN use among those with access in Boké region. **Table S2.4**: Risk factors associated with ITN use among those with access in Kindia region. **Table S2.5**: Risk factors associated with ITN use among those with access in Faranah region. **Table S2.6**: Risk factors associated with ITN use among those with access in Kankan region. **Table S2.7**: Risk factors associated with ITN use among those with access in N’zérékoré region. **Table S2.8**: Risk factors associated with ITN use among those with access in Conakry region

## Data Availability

Data used for this study is publicly available at https://dhsprogram.com/data/available-datasets.cfm and the R code for analysis is available here.

## Abbreviations

AIC: Akaike information criterion
AIDS: Acquired immunodeficiency syndrome
aOR: Adjusted odds ratio
CI: Confidence interval
DHS: Demographic and health survey
HMIS: Health management information system
ITN: Insecticide-treated net
LLIN: Long-lasting insecticide-treated net
NMCP: National malaria control programme
NGO: Non-Governmental Organization
OMVS: Senegal river basin development organization
SBCC: Social behaviour change communication
