# Supplementary material for: Factors associated with the ownership and use of insecticide-treated nets in Guinea: an analysis of the 2018 Demographic and Health Survey
Source: Malar J. 2023 Jan 26;22:29. doi: 10.1186/s12936-023-04463-z (PMC9878948; doi:10.1186/s12936-023-04463-z)
Supplement: Supplementary file 1 — Additional file 1: Table S1.1: Risk factors associated with household ITN ownership in Boké region. Table S1.2: Risk factors associated with household ITN ownership in Kindia region. Table S1.3: Risk factors associated with household ITN ownership in Faranah region. Table S1.4: Risk factors associated with household ITN ownership in Kankan region. Table S1.5: Risk factors associated with household ITN ownership in Labé region. Table S1.6: Risk factors associated with household ITN ownership in Mamou region. Table S1.7: Risk factors associated with household ITN ownership in N’zérékoré region. Table S1.8: Risk factors associated with household ITN ownership in Conakry region. Table S2.1: Risk factors associated with ITN use among those with access in Labé region. Table S2.2: Risk factors associated with ITN use among those with access in Mamou region. Table S2.3: Risk factors associated with ITN use among those with access in Boké region. Table S2.4: Risk factors associated with ITN use among those with access in Kindia region. Table S2.5: Risk factors associated with ITN use among those with access in Faranah region. Table S2.6: Risk factors associated with ITN use among those with access in Kankan region. Table S2.7: Risk factors associated with ITN use among those with access in N’zérékoré region. Table S2.8: Risk factors associated with ITN use among those with access in Conakry region [file 12936_2023_4463_MOESM1_ESM.docx]

# Supplementary Information for Diallo et al. “Factors associated to the use of insecticide-treated nets in Guinea: an analysis of the 2018 Demographic and Health Survey”

**1. Region-level analysis of risk factors associated with household ITN ownership**

Table S1.1: Risk factors associated with household ITN ownership in **Boké** region

| Explanatory variables | Univariate analysis | | Has at least one ITN versus none ITN | |
| --- | --- | --- | --- | --- |
|  | OR | 95%CI | OR adjusted | 95%CI |
| **Number of household members** | | | | |
| 1-4 | Ref |  | Ref |  |
| 5-7 | 1.48^*^ | 1.13 – 1.94 | 1.39^*^ | 1.07 – 1.86 |
| + 7 | 1.41^*^ | 1.01 – 1.95 | 1.3 | 0.83 – 2.04 |
| **Number of rooms in the household** | | | | |
| 1-3 | Ref |  | Ref |  |
| 4-6 | 1.25 | 0.91 – 1.71 | 1.18 | 0.79 – 1.79 |
| More than 7 | 1.09 | 0.52 – 2.28 | 1.09 | 0.51 – 2.36 |
| **Sex of household head** | | | | |
| Male | Ref |  | Ref |  |
| Female | 1.14 | 0.72 – 1.80 | 1.23 | 0.77 – 1.97 |
| **Marital status of the household head** | | | | |
| Married | 1.80 | 0.87 – 3.69 | 1.74 | 0.85 – 3.57 |
| Never married | Ref |  | Ref |  |
| Divorced | 1.19 | 0.34 – 4.14 | 1.16 | 0.29 – 4.6 |
| Widowed | 2.04 | 0.89 – 4.70 | 1.79 | 0.91 – 3.53 |
| **Age of household head** | | | | |
| < 30 | 0.80 | 0.40 – 1.60 |  |  |
| 30–40 | 1.11 | 0.73 – 1.69 |  |  |
| 40-50 | Ref |  |  |  |
| 50-60 | 1.25 | 0.85 – 1.83 |  |  |
| >60 | 1.16 | 0.81 – 1.65 |  |  |
| **Education level of the household head** | | | | |
| None | Ref |  | Ref |  |
| Primary | 0.82 | 0.55 – 1.21 | 0.82 | 0.54 – 1.21 |
| High | 1.38 | 1.00 – 1.90 | 1.44^*^ | 1.03 – 2.01 |
| **Presence of children under five** | | | | |
| Yes | 1.14 | 0.86 – 1.52 | 1.05 | 0.79 – 1.42 |
| No | Ref |  | Ref |  |
| **Wealth quintile** | | | | |
| Lowest | Ref |  | Ref |  |
| Second | 1.36 | 0.96 – 1.92 | 1.31 | 0.91 – 1.87 |
| Middle | 1.09 | 0.65 – 1.85 | 1.01 | 0.57 – 1.77 |
| Fourth | 1.19 | 0.61 – 2.33 | 0.82 | 0.28 – 2.38 |
| Highest | 1.47 | 0.87 – 2.46 | 0.78 | 0.26 – 2.36 |
| **Place of residence** | | | | |
| Urban | Ref |  | Ref |  |
| Rural | 0.75 | 0.47 – 1.19 | 0.63 | 0.24 – 1.7 |

Table S1.2: Risk factors associated with household ITN ownership in **Kindia** region

| Explanatory variables | Univariate analysis | | Has at least one ITN versus none ITN | |
| --- | --- | --- | --- | --- |
|  | OR | 95%CI | OR adjusted | 95%CI |
| **Number of household members** | | | | |
| 1-4 | Ref |  |  |  |
| 5-7 | 1.03 | 0.75 – 1.41 |  |  |
| + 7 | 0.97 | 0.72 – 1.31 |  |  |
| **Number of rooms in the household** | | | | |
| 1-3 | Ref |  |  |  |
| 4-6 | 0.87 | 0.63 – 1.21 |  |  |
| More than 7 | 0.68 | 0.35 – 1.33 |  |  |
| **Sex of household head** | | | | |
| Male | Ref |  | Ref |  |
| Female | 0.72 | 0.52 – 1.00 | 1.12 | 0.69 – 1.80 |
| **Marital status of the household head** | | | | |
| Married | 0.91 | 0.33 – 2.50 | 1.83 | 0.89 – 3.77 |
| Never married | Ref |  | Ref |  |
| Divorced | 0.10 | 0.01 – 1.07 | 1.18 | 0.32 – 4.24 |
| Widowed | 0.89 | 0.30 – 2.63 | 1.89 | 0.96 – 3.71 |
| **Age of household head** | | | | |
| < 30 | 1.10 | 0.67 – 1.78 |  |  |
| 30–40 | 1.00 | 0.68 – 1.46 |  |  |
| 40-50 | Ref |  |  |  |
| 50-60 | 1.32 | 0.90 – 1.95 |  |  |
| >60 | 1.39 | 0.99 – 1.96 |  |  |
| **Education level of the household head** |  |  |  |  |
| None | Ref |  |  |  |
| Primary | 1.28 | 0.81 – 2.05 |  |  |
| High | 0.86 | 0.61 – 1.21 |  |  |
| **Presence of children under five** |  |  |  |  |
| Yes | 0.92 | 0.67 – 1.27 | 1.14 | 0.86 – 1.52 |
| No | Ref |  | Ref |  |
| **Wealth quintile** |  |  |  |  |
| Lowest | Ref |  | Ref |  |
| Second | 0.50^**^ | 0.32 – 0.77 | 1.36 | 0.96 – 1.91 |
| Middle | 0.60^*^ | 0.39 – 0.91 | 1.07 | 0.62 – 1.85 |
| Fourth | 0.33^**^ | 0.20 – 0.53 | 0.92 | 0.32 – 2.65 |
| Highest | 0.33^**^ | 0.20 – 0.53 | 1.04 | 0.35 – 3.06 |
| **Place of residence** |  |  |  |  |
| Urban | Ref |  | Ref |  |
| Rural | 2.07^**^ | 1.30 – 3.30 | 0.65 | 0.23 – 1.79 |

Table S1.3: Risk factors associated with household ITN ownership in **Faranah** region

| Explanatory variables | Univariate analysis | | Has at least one ITN versus none ITN | |
| --- | --- | --- | --- | --- |
|  | OR | 95%CI | OR adjusted | 95%CI |
| **Number of household members** | | | | |
| 1-4 |  |  | Ref |  |
| 5-7 | 1.39^*^ | 1.02 – 1.88 | 1.61^**^ | 1.15 – 2.25 |
| + 7 | 1.87^**^ | 1.28 – 2.72 | 2.44^***^ | 1.54 – 3.87 |
| **Number of rooms in the household** | | | | |
| 1-3 | Ref |  | Ref |  |
| 4-6 | 1.14 | 0.76 – 1.71 | 0.83 | 0.52 – 1.32 |
| More than 7 | 0.74 | 0.32 – 1.68 | 0.42 | 0.16 – 1.06 |
| **Sex of household head** | | | | |
| Male | Ref |  |  |  |
| Female | 0.95 | 0.54 – 1.66 |  |  |
| **Marital status of the household head** | | | | |
| Married | 0.99 | 0.38 – 2.55 | 0.81 | 0.34 – 1.92 |
| Never married | Ref |  | Ref |  |
| Divorced | 1.19 | 0.30 – 4.82 | 1.44 | 0.34 – 5.95 |
| Widowed | 1.51 | 0.55 – 4.14 | 1.6 | 0.59 – 4.37 |
| **Age of household head** | | | | |
| < 30 | 0.66 | 0.40 – 1.12 |  |  |
| 30–40 | 1.20 | 0.83 – 1.76 |  |  |
| 40-50 | Ref |  |  |  |
| 50-60 | 1.05 | 0.69 – 1.59 |  |  |
| >60 | 1.03 | 0.70 – 1.52 |  |  |
| **Education level of the household head** | | | | |
| None | Ref |  | Ref |  |
| Primary | 1.20 | 0.77 – 1.88 | 1.35 | 0.82 – 2.21 |
| High | 1.52^*^ | 1.05 – 2.19 | 1.67^*^ | 1.11 – 2.5 |
| **Presence of children under five** | | | | |
| Yes | 1.36^*^ | 1.02 – 1.80 |  |  |
| No | Ref |  |  |  |
| **Wealth quintile** | | | | |
| Lowest | Ref |  | Ref |  |
| Second | 0.81 | 0.51 – 1.29 | 0.77 | 0.47 – 1.27 |
| Middle | 1.30 | 0.72 – 2.33 | 1.31 | 0.73 – 2.38 |
| Fourth | 0.77 | 0.42 – 1.39 | 0.84 | 0.38 – 1.88 |
| Highest | 1.92 | 0.75 – 4.90 | 2.11 | 0.79 – 5.64 |
| **Place of residence** | | | | |
| Urban | Ref |  | Ref |  |
| Rural | 1.17 | 0.69 – 1.99 | 1.59 | 0.88 – 2.89 |

Table S1.4: Risk factors associated with household ITN ownership in **Kankan** region

| Explanatory variables | Univariate analysis | | Has at least one ITN versus none ITN | |
| --- | --- | --- | --- | --- |
|  | OR | 95%CI | OR adjusted | 95%CI |
| **Number of household members** | | | | |
| 1-4 | Ref |  | Ref |  |
| 5-7 | 1.80^*^ | 1.18 – 2.74 | 1.52 | 0.96 – 2.40 |
| + 7 | 2.04^**^ | 1.42 – 2.91 | 1.65^*^ | 1.10 – 2.45 |
| **Number of rooms in the household** | | | | |
| 1-3 | Ref |  | Ref |  |
| 4-6 | 1.07 | 0.74 – 1.56 |  |  |
| More than 7 | 1.84 | 0.86 – 3.91 |  |  |
| **Sex of household head** | | | | |
| Male | Ref |  |  |  |
| Female | 0.62 | 0.30 – 1.30 |  |  |
| **Age of household head** | | | | |
| < 30 | 0.72 | 0.48 – 1.09 |  |  |
| 30–40 | 1.01 | 0.63 – 1.62 |  |  |
| 40-50 | Ref |  |  |  |
| 50-60 | 1.02 | 0.64 – 1.61 |  |  |
| >60 | 0.60 | 0.35 – 1.03 |  |  |
| **Education level of the household head** | | | | |
| None | Ref |  |  |  |
| Primary | 1.04 | 0.58 – 1.87 |  |  |
| High | 1.10 | 0.71 – 1.73 |  |  |
| **Presence of children under five** | | | | |
| Yes | 2.04^**^ | 1.38 – 3.01 | 1.68^*^ | 1.08 – 2.59 |
| No | Ref |  | Ref |  |
| **Wealth quintile** | | | | |
| Lowest | Ref |  |  |  |
| Second | 1.05 | 0.57 – 1.91 |  |  |
| Middle | 1.24 | 0.78 – 1.97 |  |  |
| Fourth | 1.04 | 0.52 – 2.09 |  |  |
| Highest | 0.82 | 0.42 – 1.60 |  |  |
| **Place of residence** | | | | |
| Urban | Ref |  |  |  |
| Rural | 1.21 | 0.70 – 2.07 |  |  |

Table S1.5: Risk factors associated with household ITN ownership in **Labé** region.

| Explanatory variables | Univariate analysis | | Has at least one ITN versus none ITN | |
| --- | --- | --- | --- | --- |
|  | OR | 95%CI | OR adjusted | 95%CI |
| **Number of household members** | | | | |
| 1-4 | Ref |  |  |  |
| 5-7 | 0.96 | 0.75 – 1.21 |  |  |
| + 7 | 1.14 | 0.83 – 1.58 |  |  |
| **Number of rooms in the household** | | | | |
| 1-3 | Ref |  |  |  |
| 4-6 | 1.34 | 0.99 – 1.82 |  |  |
| More than 7 | 1.48 | 0.47 – 4.65 |  |  |
| **Sex of household head** | | | | |
| Male | Ref |  |  |  |
| Female | 0.83 | 0.63 – 1.08 |  |  |
| **Marital status of the household head** | | | | |
| Married | 3.91 | 0.85 – 10.64 |  |  |
| Never married | Ref |  |  |  |
| Divorced | 2.84 | 0.66 – 12.20 |  |  |
| Widowed | 3.91^*^ | 1.01 – 14.75 |  |  |
| **Age of household head** | | | | |
| < 30 | 0.59 | 0.30 – 1.16 | 0.69 | 0.47 – 1.02 |
| 30–40 | 0.65 | 0.41 – 1.05 | 1.00 | 0.63 – 1.61 |
| 40-50 | Ref |  | Ref |  |
| 50-60 | 1.12 | 0.75 – 1.67 | 0.97 | 0.62 – 1.52 |
| >60 | 1.22 | 0.85 – 1.74 | 0.57^*^ | 0.33 – 0.99 |
| **Education level of the household head** | | | | |
| None | Ref |  |  |  |
| Primary | 1.25 | 0.79 – 1.98 |  |  |
| High | 1.57^*^ | 1.02 – 2.43 |  |  |
| **Presence of children under five** | | | | |
| Yes | 0.70^*^ | 0.55 – 0.89 |  |  |
| No | Ref |  |  |  |
| **Wealth quintile** | | | | |
| Lowest | Ref |  | Ref |  |
| Second | 0.95 | 0.65 – 1.40 | 1.01 | 0.54 – 1.91 |
| Middle | 1.45 | 0.95 – 2.20 | 1.24 | 0.77 – 2.01 |
| Fourth | 2.43^**^ | 1.62 – 3.62 | 1.00 | 0.51 – 2.01 |
| Highest | 4.31^**^ | 1.93 – 9.62 | 0.77 | 0.38 – 1.53 |
| **Place of residence** | | | | |
| Urban | Ref |  |  |  |
| Rural | 0.55^*^ | 0.37 – 0.82 |  |  |

Table S1.6: Risk factors associated with household ITN ownership in **Mamou** region

| Explanatory variables | Univariate analysis | | Has at least one ITN versus none ITN | |
| --- | --- | --- | --- | --- |
|  | OR | 95%CI | OR adjusted | 95%CI |
| **Number of household members** | | | | |
| 1-4 | Ref |  |  |  |
| 5-7 | 1.10 | 0.83 – 1.45 |  |  |
| + 7 | 1.32 | 0.84 – 2.07 |  |  |
| **Number of rooms in the household** | | | | |
| 1-3 | Ref |  | Ref |  |
| 4-6 | 0.95 | 0.69 – 1.30 | 0.81 | 0.59 – 1.11 |
| More than 7 | 6.86 | 0.78 – 60.39 | 5.72 | 0.58 – 56.05 |
| **Sex of household head** | | | | |
| Male | Ref |  | Ref |  |
| Female | 0.76 | 0.56 – 1.04 | 0.75 | 0.54 – 1.03 |
| **Marital status of the household head** | | | | |
| Married | 0.34 | 0.03 – 4.10 |  |  |
| Never married | Ref |  |  |  |
| Divorced | 0.59 | 0.04 – 7.82 |  |  |
| Widowed | 0.24 | 0.02 – 3.30 |  |  |
| **Age of household head** | | | | |
| < 30 | 0.97 | 0.53 – 1.79 |  |  |
| 30–40 | 1.38 | 0.89 – 2.14 |  |  |
| 40-50 | Ref |  |  |  |
| 50-60 | 0.91 | 0.61 – 1.37 |  |  |
| >60 | 1.27 | 0.85 – 1.89 |  |  |
| **Education level of the household head** | | | | |
| None | Ref |  |  |  |
| Primary | 1.75 | 1.07 – 2.87 |  |  |
| High | 1.55 | 0.90 – 2.65 |  |  |
| **Presence of children under five** | | | | |
| Yes | 1.00 | 0.77 – 1.30 |  |  |
| No | Ref |  |  |  |
| **Wealth quintile** | | | | |
| Lowest | Ref |  | Ref |  |
| Second | 0.93 | 0.55 – 1.59 | 0.96 | 0.56 – 1.63 |
| Middle | 1.21 | 0.75 – 1.96 | 1.28 | 0.79 – 2.15 |
| Fourth | 1.40 | 0.88 – 2.25 | 1.73^*^ | 1.06 – 2.82 |
| Highest | 2.01 | 0.92 – 4.41 | 2.98^**^ | 1.49 – 5.96 |
| **Place of residence** | | | | |
| Urban | Ref |  | Ref |  |
| Rural | 0.88 | 0.44 – 1.76 | 0.9 | 0.38 – 2.16 |

Table S1.7: Risk factors associated with household ITN ownership in **N’zérékoré** region

| Explanatory variables | Univariate analysis | | Has at least one ITN versus none ITN | |
| --- | --- | --- | --- | --- |
|  | OR | 95%CI | OR adjusted | 95%CI |
| **Number of household members** | | | | |
| 1-4 | Ref |  | Ref |  |
| 5-7 | 0.89 | 0.58 – 1.37 | 0.84 | 0.54 – 1.29 |
| + 7 | 1.00 | 0.62 – 1.62 | 0.97 | 0.59 – 1.59 |
| **Number of rooms in the household** | | | | |
| 1-3 | Ref |  |  |  |
| 4-6 | 1.01 | 0.68 – 1.50 |  |  |
| More than 7 | 1.12 | 0.39 – 3.22 |  |  |
| **Sex of household head** | | | | |
| Male | Ref |  |  |  |
| Female | 0.82 | 0.56 – 1.22 |  |  |
| **Marital status of the household head** | | | | |
| Married | 2.63^*^ | 1.09 – 6.39 |  |  |
| Never married | Ref |  |  |  |
| Divorced | 2.42 | 0.83 – 7.10 |  |  |
| Widowed | 1.89 | 0.70 – 5.07 |  |  |
| **Age of household head** | | | | |
| < 30 | 0.83 | 0.51 – 1.35 |  |  |
| 30–40 | 1.26 | 0.90 – 1.78 |  |  |
| 40-50 | Ref |  |  |  |
| 50-60 | 0.98 | 0.63 – 1.50 |  |  |
| >60 | 0.73 | 0.49 – 1.10 |  |  |
| **Education level of the household head** | | | | |
| None | Ref |  | Ref |  |
| Primary | 0.88 | 0.57 – 1.37 | 0.86 | 0.55 – 1.33 |
| High | 1.49^*^ | 1.01 – 2.23 | 1.53^*^ | 1.01 – 2.31 |
| **Presence of children under five** | | | | |
| Yes | 1.14 | 0.86 – 1.51 |  |  |
| No | Ref |  |  |  |
| **Wealth quintile** | | | | |
| Lowest |  |  | Ref |  |
| Second | 1.21 | 0.81 – 1.80 | 1.19 | 0.78 – 1.82 |
| Middle | 1.12 | 0.70 – 1.79 | 1.07 | 0.65 – 1.77 |
| Fourth | 0.95 | 0.59 – 1.54 | 0.84 | 0.39 – 1.83 |
| Highest | 1.96 | 0.77 – 4.96 | 1.60 | 0.48 – 5.37 |
| **Place of residence** | | | | |
| Urban | Ref |  | Ref |  |
| Rural | 1.03 | 0.56 – 1.87 | 1.02 | 0.46 – 2.25 |

Table S1.8: Risk factors associated with household ITN ownership in Conakry region

| Explanatory variables | Univariate analysis | | Has at least one ITN versus none ITN | |
| --- | --- | --- | --- | --- |
|  | OR | 95%CI | OR adjusted | 95%CI |
| **Number of household members** | | | | |
| 1-4 | Ref |  | Ref |  |
| 5-7 | 1.48^*^ | 1.13 – 1.94 | 0.84 | 0.56 – 1.26 |
| + 7 | 1.41^*^ | 1.01 – 1.95 | 0.83 | 0.48 – 1.44 |
| **Number of rooms in the household** | | | | |
| 1-3 | Ref |  |  |  |
| 4-6 | 1.25 | 0.91 – 1.71 |  |  |
| More than 7 | 1.09 | 0.52 – 1.71 |  |  |
| **Sex of household head** | | | | |
| Male | Ref |  |  |  |
| Female | 1.14 | 0.72 – 1.80 |  |  |
| **Marital status of the household head** | | | | |
| Married | 1.80 | 0.87 – 3.69 |  |  |
| Never married | Ref |  |  |  |
| Divorced | 1.19 | 0.34 – 4.14 |  |  |
| Widowed | 2.04 | 0.89 – 4.70 |  |  |
| **Age of household head** | | | | |
| < 30 | 0.80 | 0.40 – 1.60 | 0.34^**^ | 0.17 – 0.67 |
| 30–40 | 1.11 | 0.73 – 1.69 | 0.78 | 0.51 – 1.22 |
| 40-50 | Ref |  | Ref |  |
| 50-60 | 1.25 | 0.85 – 1.83 | 0.51^**^ | 0.32 – 0.79 |
| >60 | 1.16 | 0.81 – 1.65 | 0.61^*^ | 0.41 – 0.93 |
| **Education level of the household head** | | | | |
| None | Ref |  |  |  |
| Primary | 0.82 | 0.55 – 1.21 |  |  |
| High | 1.38 | 1.00 – 1.90 |  |  |
| **Presence of children under five** | | | | |
| Yes | 1.14 | 0.86 – 1.52 |  |  |
| No | Ref |  |  |  |
| **Wealth quintile** | | | | |
| Lowest | Ref |  |  |  |
| Second | 1.36 | 0.96 – 1.92 |  |  |
| Middle | 1.09 | 0.65 – 1.85 |  |  |
| Fourth | 1.19 | 0.61 – 2.33 |  |  |
| Highest | 1.47 | 0.87 – 2.46 |  |  |

**2. Region-level analysis of risk factors associated with ITN use among those with access**

Table S2.1: Risk factors associated with ITN use among those with access in **Labé** region

| Explanatory variables | Univariate Analysis | | Multivariate analysis | |
| --- | --- | --- | --- | --- |
|  | OR | 95%CI | OR | 95%CI |
| **Number of household members** | | | | |
| 1-4 | 1.17 | 0.70 – 1.94 |  |  |
| 5-7 | 1.15 | 0.78 – 1.77 |  |  |
| More than 7 | Ref |  | Ref |  |
| **Sex** | | | | |
| Male | Ref |  |  |  |
| Female | 1.07 | 0.88 – 1.31 |  |  |
| **Numbers of rooms in the household** | | | | |
| 1-3 | Ref |  | Ref |  |
| 4-6 | 0.48 | 0.31 – 0.76 | 0.42^**^ | 0.25 – 0.70 |
| More than 7 | 1.76 | 0.40 – 7.67 | 1.23 | 0.27 – 5.67 |
| **Education** |  |  |  |  |
| None | 0.60^*^ | 0.37 – 0.95 | 0.67 | 0.37 – 1.25 |
| Primary | 0.53^*^ | 0.34 – 0.83 | 0.80 | 0.45 – 1.43 |
| High | Ref |  | Ref |  |
| **Age of household members** | | | | |
| 0-5 | Ref |  | Ref |  |
| 5-10 | 0.73^*^ | 0.58 – 0.92 |  |  |
| 10-20 | 0.89 | 0.64 – 1.23 |  |  |
| 20-40 | 2.19^***^ | 1.76 – 2.74 |  |  |
| 40-50 | 2.23^***^ | 1.57 – 3.18 |  |  |
| 50-60 | 1.53^*^ | 1.06 – 2.20 |  |  |
| + 60 | 1.61^*^ | 1.05 – 2.45 |  |  |
| **Age of household head** |  |  |  |  |
| < 30 | 1.25 | 0.64 – 2.42 | 1.02 | 0.51 – 2.11 |
| 30 - 40 | 1.31 | 0.58 – 2.97 | 1.15 | 0.51 – 2.63 |
| 40 - 50 | Ref |  | Ref |  |
| 50 - 60 | 0.58 | 0.33 – 1.02 | 0.68 | 0.39 – 1.21 |
| + 60 | 0.75 | 0.41 – 1.36 | 0.86 | 0.48 – 1.54 |
| **Wealth quintile** | | | | |
| Lowest | Ref |  | Ref |  |
| Second | 0.94 | 0.51 – 1.72 |  |  |
| Middle | 0.92 | 0.55 – 1.52 |  |  |
| Fourth | 1.74 | 0.87 – 3.46 |  |  |
| Highest | 1.42 | 0.56 – 3.56 |  |  |
| **Marital status** | | | | |
| Married | 3.32^***^ | 1.85 – 5.98 | 3.91^***^ | 2.11 – 7.24 |
| Never Married | Ref |  | Ref |  |
| Divorced | 2.36 | 0.75 – 7.41 | 2.34 | 0.76 – 7.14 |
| Widowed | 1.24 | 0.56 – 2.75 | 1.59 | 0.65 – 3.85 |
| **Presence of children under five** | | | | |
| Yes | 1.52 | 1.05 – 2.21 | 1.86^*^ | 1.16 – 3.1 |
| No | Ref |  | Ref |  |
| **Pregnancy status** | | | | |
| Yes | Ref |  | Ref |  |
| No | 1.99^*^ | 1.07 – 3.68 |  |  |
| **Place of residence** | | | | |
| Urban | Ref |  | Ref |  |
| Rural | 1.52^*^ | 1.05 – 2.21 | 0.42^*^ | 0.18 – 1.00 |

Table S2.2: Risk factors associated with ITN use among those with access in **Mamou** region

| Explanatory variables | Univariate Analysis | | Multivariate analysis | |
| --- | --- | --- | --- | --- |
|  | OR | 95%CI | OR | 95%CI |
| **Number of household members** | | | | |
| 1-4 | 1.28 | 0.81 – 2.04 | 0.73 | 0.45 – 1.20 |
| 5-7 | 1.06 | 0.63 – 1.79 | 0.78 | 0.46 – 1.32 |
| More than 7 | Ref |  | Ref |  |
| **Sex** |  |  |  |  |
| Male | Ref |  |  |  |
| Female | 1.19 | 0.96 – 1.47 |  |  |
| **Numbers of rooms in the household** | | | | |
| 1-3 | Ref |  | Ref |  |
| 4-6 | 1.02 | 0.63 – 1.65 | 0.88 | 0.52 – 1.48 |
| More than 7 | 0.34 | 0.09 – 1.28 | 0.32^*^ | 0.13 – 0.78 |
| **Education** | | | | |
| None | 1.18 | 0.72 – 1.93 |  |  |
| Primary | 0.68 | 0.38 – 1.20 |  |  |
| High | Ref |  | Ref |  |
| **Age of household members** | | | | |
| 0-5 | Ref |  | Ref |  |
| 5-10 | 0.77 | 0.53 – 1.12 |  |  |
| 10-20 | 0.88 | 0.64 – 1.20 |  |  |
| 20-40 | 3.43^***^ | 2.43 – 4.84 |  |  |
| 40-50 | 2.61^***^ | 1.70 – 3.99 |  |  |
| 50-60 | 3.20^***^ | 2.06 – 4.96 |  |  |
| + 60 | 2.50^***^ | 1.57 – 3.98 |  |  |
| **Age of household head** | | | | |
| < 30 | 1.71 | 0.57 – 5.15 | 1.55 | 0.49 – 4.88 |
| 30 - 40 | 1.32 | 0.72 – 2.42 | 1.28 | 0.69 – 2.35 |
| 40 - 50 | Ref |  | Ref |  |
| 50 - 60 | 1.21 | 0.68 – 2.16 | 1.11 | 0.60 – 2.06 |
| + 60 | 0.73 | 0.44 – 1.21 | 0.59 | 0.34 – 0.99 |
| **Wealth quintile** | | | | |
| Lowest | Ref |  | Ref |  |
| Second | 1.56 | 0.96 – 2.52 | 1.68 | 0.97 – 2.91 |
| Middle | 1.34 | 0.76 – 2.35 | 1.41 | 0.82 – 2.41 |
| Fourth | 1.32 | 0.58 – 2.97 | 2.1 | 0.95 – 4.22 |
| Highest | 2.72^***^ | 1.44 – 5.12 | 4.93^**^ | 1.86 – 13.1 |
| **Marital status** | | | | |
| Married | 5.38^***^ | 3.47 – 8.32 | 5.89^***^ | 3.97 – 8.73 |
| Never Married | Ref |  | Ref |  |
| Divorced | 4.5^*^ | 1.89 – 16.3 | 2.31^*^ | 1.70 – 5.78 |
| Widowed | 3.14^***^ | 1.76 – 5.61 | 3.58^***^ | 1.96 – 6.55 |
| **Presence of children under five** | | | | |
| Yes | 0.89 | 0.57 – 1.39 |  |  |
| No | Ref |  |  |  |
| **Pregnancy status** | | | | |
| Yes | Ref |  |  |  |
| No | 1.07 | 0.43 – 2.65 |  |  |
| **Place of residence** | | | | |
| Urban | Ref |  | Ref |  |
| Rural | 1.20 | 0.47 – 3.06 | 1.78 | 0.66 – 4.79 |

Table S2.3: Risk factors associated with ITN use among those with access in **Boké** region

| Explanatory variables | Univariate Analysis | | Multivariate analysis | |
| --- | --- | --- | --- | --- |
|  | OR | 95%CI | OR | 95%CI |
| **Number of household members** | | | | |
| 1-4 | 1.60 | 1.00 – 2.55 | 1.41 | 0.85 – 2.33 |
| 5-7 | 1.57^*^ | 1.06 – 2.33 | 1.55 | 0.96 – 2.49 |
| More than 7 | Ref |  | Ref |  |
| **Sex** | | | | |
| Male | Ref |  | Ref |  |
| Female | 0.99 | 0.85 – 1.16 | 0.86 | 0.71 – 1.03 |
| **Numbers of rooms in the household** | | | | |
| 1-3 | Ref |  | Ref |  |
| 4-6 | 0.80 | 0.55 – 1.16 | 1.04 | 0.66 – 1.63 |
| More than 7 | 1.21 | 0.67 – 2.20 | 1.66 | 0.72 – 3.83 |
| **Education** | | | | |
| None | 0.83 | 0.55 – 1.25 |  |  |
| Primary | 0.68^*^ | 0.49 – 0.95 |  |  |
| High | Ref |  | Ref |  |
| **Age of household members** | | | | |
| 0-5 | Ref |  | Ref |  |
| 5-10 | 0.69^***^ | 0.56 – 0.84 | 0.68^**^ | 0.54 – 0.85 |
| 10-20 | 0.73^*^ | 0.57 – 0.93 | 0.66^*^ | 0.49 – 0.89 |
| 20-40 | 1.40^*^ | 1.11 – 1.77 | 0.72 | 0.43 – 1.22 |
| 40-50 | 2.11^***^ | 1.49 – 3.00 | 0.93 | 0.51 – 1.69 |
| 50-60 | 1.98^***^ | 1.31 – 2.98 | 0.92 | 0.46 – 1.81 |
| + 60 | 1.67^***^ | 1.21 – 2.31 | 0.87 | 0.43 – 1.74 |
| **Age of household head** | | | | |
| < 30 | 1.04 | 0.56 – 1.96 |  |  |
| 30 - 40 | 1.01 | 0.64 – 1.60 |  |  |
| 40 - 50 | Ref |  | Ref |  |
| 50 - 60 | 0.90 | 0.51 – 1.60 |  |  |
| + 60 | 0.89 | 0.51 – 1.55 |  |  |
| **Wealth quintile** | | | | |
| Lowest | Ref |  | Ref |  |
| Second | 0.86 | 0.50 – 1.46 | 0.86 | 0.51 – 1.46 |
| Middle | 0.75 | 0.40 – 1.41 | 0.77 | 0.41 – 1.44 |
| Fourth | 1.46 | 0.79 – 2.67 | 1.32 | 0.64 – 2.75 |
| Highest | 1.24 | 0.59 – 2.61 | 1.13 | 0.43 – 2.61 |
| **Marital status** | | | | |
| Married | 2.90^***^ | 2.12 – 3.97 |  |  |
| Never Married | Ref |  | Ref |  |
| Divorced | 1.22 | 0.56 – 2.68 |  |  |
| Widowed | 1.54 | 0.93 – 2.57 |  |  |
| **Presence of children under five** | | | | |
| Yes | 1.02 | 0.74 – 1.43 |  |  |
| No | Ref |  | Ref |  |
| **Pregnancy status** | | | | |
| Yes | Ref |  | Ref |  |
| No | 0.77 | 0.46 – 1.30 |  |  |
| **Place of residence** | | | | |
| Urban | Ref |  | Ref |  |
| Rural | 0.64 | 0.36 – 1.15 | 0.75 | 0.39 – 1.45 |

Table S2.4: Risk factors associated with ITN use among those with access in **Kindia** region

| Explanatory variables | Univariate Analysis | | Multivariate analysis | |
| --- | --- | --- | --- | --- |
|  | OR | 95%CI | OR | 95%CI |
| **Number of household members** | | | | |
| 1-4 | 0.92 | 0.56 – 1.52 | 0.84 | 0.51 – 1.39 |
| 5-7 | 1.22 | 0.73 – 2.05 | 1.20 | 0.72 – 1.99 |
| More than 7 | Ref |  | Ref |  |
| **Sex** | | | | |
| Male | Ref |  | Ref |  |
| Female | 1.21 | 0.98 – 1.49 |  |  |
| **Numbers of rooms in the household** | | | | |
| 1-3 | Ref |  | Ref |  |
| 4-6 | 1.10 | 0.78 – 1.56 |  |  |
| More than 7 | 1.15 | 0.54 – 2.47 |  |  |
| **Education** | | | | |
| None | 0.69 | 0.42 – 1.13 |  |  |
| Primary | 0.58^*^ | 0.38 – 0.88 |  |  |
| High | Ref |  | Ref |  |
| **Age of household members** | | | | |
| 0-5 | Ref |  | Ref |  |
| 5-10 | 0.87 | 0.67 – 1.12 |  |  |
| 10-20 | 0.89 | 0.64 – 1.23 |  |  |
| 20-40 | 2.19^***^ | 1.59 – 3.00 |  |  |
| 40-50 | 2.58^***^ | 1.60 – 4.17 |  |  |
| 50-60 | 1.79^*^ | 1.02 – 3.17 |  |  |
| + 60 | 1.27 | 0.83 – 1.94 |  |  |
| **Age of household head** | | | | |
| < 30 | 0.94 | 0.48 – 1.84 | 0.96 | 0.48 – 1.92 |
| 30 - 40 | 0.98 | 0.59 – 1.64 | 1.07 | 0.63 – 1.82 |
| 40 - 50 | Ref |  | Ref |  |
| 50 - 60 | 0.89 | 0.54 – 1.45 | 0.98 | 0.58 – 1.66 |
| + 60 | 0.59^*^ | 0.38 – 0.92 | 0.60 | 0.36 – 1.0 |
| **Wealth quintile** | | | | |
| Lowest | Ref |  | Ref |  |
| Second | 1.63 | 0.92 – 2.89 | 1.58 | 0.89 – 2.78 |
| Middle | 2.10^*^ | 1.05 – 4.18 | 2.10^*^ | 1.01 – 4.35 |
| Fourth | 2.01^*^ | 1.15 – 3.51 | 1.71 | 0.79 – 3.67 |
| Highest | 2.56^*^ | 1.37 – 4.81 | 1.99 | 0.84 – 4.72 |
| **Marital status** | | | | |
| Married | 2.60^***^ | 1.88 – 3.60 | 3.02^***^ | 2.26 – 4.04 |
| Never Married | Ref |  | Ref |  |
| Divorced | 2.93 | 0.77 – 11.64 | 3.15 | 0.98 – 10.1 |
| Widowed | 1.69 | 0.88 – 3.22 | 2.05^*^ | 1.13 – 3.74 |
| **Presence of children under five** | | | | |
| Yes | 1.11 | 0.75 – 1.64 |  |  |
| No | Ref |  | Ref |  |
| **Pregnancy status** | | | | |
| Yes | Ref |  | Ref |  |
| No | 0.93 | 0.28 – 3.14 |  |  |
| **Place of residence** | | | | |
| Urban | Ref |  | Ref |  |
| Rural | 0.61 | 0.34 – 1.10 | 0.69 | 0.27 – 3.70 |

Table S2.5: Risk factors associated with ITN use among those with access in **Faranah** region

| Explanatory variables | Univariate Analysis | | Multivariate analysis | |
| --- | --- | --- | --- | --- |
|  | OR | 95%CI | OR | 95%CI |
| **Number of household members** | | | | |
| 1-4 | 1.19 | 0.57 – 2.46 |  |  |
| 5-7 | 1.14 | 0.80 – 1.63 |  |  |
| More than 7 | Ref |  | Ref |  |
| **Sex** | | | | |
| Male | Ref |  | Ref |  |
| Female | 1.58^***^ | 1.30 – 1.91 | 1.44^**^ | 1.15 – 1.78 |
| **Numbers of rooms in the household** | | | | |
| 1-3 | Ref |  | Ref |  |
| 4-6 | 0.81 | 0.58 – 1.12 | 0.86 | 0.61 – 1.23 |
| More than 7 | 0.56 | 0.27 – 1.18 | 0.57 | 0.24 – 1.35 |
| **Education** | | | | |
| None | 0.95 | 0.60 – 1.49 |  |  |
| Primary | 0.66 | 0.42 – 1.06 |  |  |
| High | Ref |  | Ref |  |
| **Age of household members** | | | | |
| 0-5 | Ref |  | Ref |  |
| 5-10 | 0.54^***^ | 0.43 – 0.69 | 0.52^***^ | 0.41 – 0.66 |
| 10-20 | 0.69^*^ | 0.52 – 0.91 | 0.69 | 0.46 – 1.04 |
| 20-40 | 1.82^***^ | 1.33 – 2.48 | 0.97 | 0.46 – 2.03 |
| 40-50 | 2.35^*^ | 1.23 – 4.49 | 1.21 | 0.37 – 4.0 |
| 50-60 | 3.20^***^ | 1.77 – 5.81 | 1.85 | 0.54 – 6.36 |
| + 60 | 1.73 | 0.98 – 3.06 | 1.18 | 0.35 – 3.98 |
| **Age of household head** | | | | |
| < 30 | 0.72 | 0.32 – 1.61 |  |  |
| 30 - 40 | 0.85 | 0.45 – 1.59 |  |  |
| 40 - 50 | Ref |  | Ref |  |
| 50 - 60 | 0.81 | 0.51 – 1.31 |  |  |
| + 60 | 0.73 | 0.41 – 1.28 |  |  |
| **Wealth quintile** | | | | |
| Lowest | Ref |  | Ref |  |
| Second | 0.73 | 0.41 – 1.28 | 0.68 | 0.36 – 1.32 |
| Middle | 0.99 | 0.59 – 1.69 | 1.01 | 0.57 – 1.79 |
| Fourth | 1.09 | 0.61 – 1.96 | 1.1 | 0.39 – 2.57 |
| Highest | 0.84 | 0.34 – 2.06 | 0.71 | 0.24 – 2.1 |
| **Marital status** | | | | |
| Married | 4.92^***^ | 2.97 – 8.15 | 3.45^**^ | 1.53 – 7.78 |
| Never Married | Ref |  | Ref |  |
| Divorced | 2.47 | 0.55 – 11.05 | 1.87 | 0.35 – 9.95 |
| Widowed | 2.24 | 0.95 – 5.26 | 1.14 | 0.37 – 3.51 |
| **Presence of children under five** | | | | |
| Yes | 1.07 | 0.69 – 1.65 |  |  |
| No | Ref |  | Ref |  |
| **Pregnancy status** | | | | |
| Yes | Ref |  | Ref |  |
| No | 2.44^***^ | 1.51 – 3.95 |  |  |
| **Place of residence** | | | | |
| Urban | Ref |  | Ref |  |
| Rural | 0.72 | 0.42 – 1.22 | 0.56 | 0.25 – 1.29 |

Table S2.6: Risk factors associated with ITN use among those with access in **Kankan** region

| Explanatory variables | Univariate Analysis | | Multivariate analysis | |
| --- | --- | --- | --- | --- |
|  | OR | 95%CI | OR | 95%CI |
| **Number of household members** | | | | |
| 1-4 | 0.99 | 0.71 – 1.38 | 2.03 | 0.77 – 5.35 |
| 5-7 | 1.20 | 0.88 – 1.64 | 2.14 | 0.97 – 4.70 |
| More than 7 | Ref |  | Ref |  |
| **Sex** | | | | |
| Male | Ref |  | Ref |  |
| Female | 1.31^***^ | 1.12 – 1.52 |  |  |
| **Numbers of rooms in the household** | | | | |
| 1-3 | Ref |  | Ref |  |
| 4-6 | 0.69^*^ | 0.49 – 0.97 | 0.68 | 0.39 – 1.19 |
| More than 7 | 1.23 | 0.58 – 2.62 | 3.30 | 0.76 – 14.4 |
| **Education** | | | | |
| None | 0.99 | 0.70 – 1.41 |  |  |
| Primary | 0.86 | 0.63 – 1.16 |  |  |
| High | Ref |  | Ref |  |
| **Age of household members** | | | | |
| 0-5 | Ref |  | Ref |  |
| 5-10 | 0.61^***^ | 0.49 – 0.75 | 0.99 | 0.63 – 1.56 |
| 10-20 | 0.46^***^ | 0.37 – 0.57 | 1.17 | 0.67 – 2.04 |
| 20-40 | 1.01 | 0.87 – 1.17 | 3.10^*^ | 1.31 – 7.29 |
| 40-50 | 1.04 | 0.79 – 1.37 | 4.52 | 2.76 – 12.3 |
| 50-60 | 0.80 | 0.58 – 1.09 | 2.29 | 0.65 – 8.08 |
| + 60 | 0.69 | 0.45 – 1.05 | 4.12 | 0.89 – 19.1 |
| **Age of household head** |  |  |  |  |
| < 30 | 1.17 | 0.71 – 1.93 | 1.15 | 0.29 – 4.64 |
| 30 - 40 | 1.52 | 0.96 – 2.41 | 3.15^**^ | 1.48 – 6.67 |
| 40 - 50 | Ref |  | Ref |  |
| 50 - 60 | 0.95 | 0.63 – 1.43 | 1.61 | 0.78 – 3.30 |
| + 60 | 0.66 | 0.39 – 1.11 | 0.53 | 0.21 – 1.32 |
| **Wealth quintile** |  |  |  |  |
| Lowest | Ref |  |  |  |
| Second | 0.98 | 0.63 – 1.53 |  |  |
| Middle | 0.96 | 0.64 – 1.42 |  |  |
| Fourth | 1.03 | 0.61 – 1.72 |  |  |
| Highest | 0.67 | 0.35 – 1.31 |  |  |
| **Marital status** |  |  |  |  |
| Married | 2.92^***^ | 1.91 – 4.47 | 2.11 | 0.89 – 5.02 |
| Never Married | Ref |  | Ref |  |
| Divorced | 5.7^**^ | 1.98 – 16.39 | 2.1 | 0.78 – 5.74 |
| Widowed | 2.26^*^ | 1.13 – 4.52 | 0.39 | 0.07 – 2.29 |
| **Presence of children under five** | | | | |
| Yes | 1.48^*^ | 1.01 – 2.16 |  |  |
| No | Ref |  |  |  |
| **Pregnancy status** | | | | |
| Yes | Ref |  |  |  |
| No | 0.94 | 0.63 – 1.41 |  |  |
| **Place of residence** | | | | |
| Urban | Ref |  | Ref |  |
| Rural | 1.19 | 0.67 – 2.10 | 0.69 | 0.38 – 1.25 |

Table S2.7: Risk factors associated with ITN use among those with access in **N’zérékoré** region

| Explanatory variables | Univariate Analysis | | Multivariate analysis | |
| --- | --- | --- | --- | --- |
|  | OR | 95%CI | OR | 95%CI |
| **Number of household members** | | | | |
| 1-4 | 1.58 | 0.80 – 3.14 |  |  |
| 5-7 | 1.10 | 0.73 – 1.65 |  |  |
| More than 7 | Ref |  |  |  |
| **Sex** |  |  |  |  |
| Male | Ref |  |  |  |
| Female | 0.81 | 0.58 – 1.12 |  |  |
| **Numbers of rooms in the household** | | | | |
| 1-3 | Ref |  |  |  |
| 4-6 | 0.80 | 0.45 – 1.42 |  |  |
| More than 7 | 0.86 | 0.39 – 1.92 |  |  |
| **Education** | | | | |
| None | 0.84 | 0.47 – 1.50 | 0.74 | 0.37 – 1.48 |
| Primary | 0.85 | 0.49 – 1.46 | 1.13 | 0.63 – 1.96 |
| High | Ref |  | Ref |  |
| **Age of household members** | | | | |
| 0-5 | Ref |  | Ref |  |
| 5-10 | 0.63^*^ | 0.41 – 0.96 | 0.45^**^ | 0.26 – 0.79 |
| 10-20 | 0.56^*^ | 0.37 – 0.84 | 0.51^*^ | 0.29 – 0.88 |
| 20-40 | 1.20 | 0.78 – 1.84 | 0.81 | 0.37 – 1.73 |
| 40-50 | 3.14^*^ | 1.22 – 8.06 | 2.57 | 0.61 – 10.8 |
| 50-60 | 1.58 | 0.76 – 3.29 | 1.74 | 0.52 – 5.87 |
| + 60 | 1.33 | 0.59 – 3.01 | 2.05 | 0.63 – 6.69 |
| **Age of household head** | | | | |
| < 30 | 0.75 | 0.27 – 2.06 | 0.76 | 0.27 – 2.14 |
| 30 - 40 | 1.17 | 0.64 – 2.12 | 1.24 | 0.67 – 2.29 |
| 40 - 50 | Ref |  | Ref |  |
| 50 - 60 | 0.69 | 0.36 – 1.31 | 0.75 | 0.39 – 1.41 |
| + 60 | 0.84 | 0.48 – 1.49 | 0.71 | 0.37 – 1.33 |
| **Wealth quintile** | | | | |
| Lowest | Ref |  | Ref |  |
| Second | 1.03 | 0.63 – 1.69 | 0.95 | 0.56 – 1.61 |
| Middle | 1.92^*^ | 1.01 – 3.64 | 1.97^*^ | 1.04 – 3.73 |
| Fourth | 1.65 | 0.63 – 4.28 | 1.55 | 0.55 – 4.36 |
| Highest | 2.10 | 0.66 – 6.72 | 1.88 | 0.48 – 7.26 |
| **Marital status** | | | | |
| Married | 4.45 | 2.78 – 7.13 | 2.78^**^ | 1.42 – 5.42 |
| Never Married | Ref |  | Ref |  |
| Divorced | 0.67 | 0.41 – 1.09 | 0.40^*^ | 0.18 – 0.89 |
| Widowed | 1.46 | 0.73 – 2.89 | 056 | 0.20 – 1.54 |
| **Presence of children under five** | | | | |
| Yes | 0.91 | 0.59 – 1.41 | 0.68 | 0.44 – 1.1 |
| No | Ref |  | Ref |  |
| **Pregnancy status** | | | | |
| Yes | Ref |  |  |  |
| No | 0.83 | 0.35 – 1.96 |  |  |
| **Place of residence** | | | | |
| Urban | Ref |  |  |  |
| Rural | 0.80 | 0.47 – 1.33 |  |  |

Table S2.8: Risk factors associated with ITN use among those with access in **Conakry** region

| Explanatory variables | Univariate Analysis | | Multivariate analysis | |
| --- | --- | --- | --- | --- |
|  | OR | 95%CI | OR | 95%CI |
| **Number of household members** | | | | |
| 1-4 | 1.97 | 0.91 – 4.25 | 0.87 | 0.33 – 2.30 |
| 5-7 | 0.92 | 0.43 – 1.96 | 0.57 | 0.25 – 1.32 |
| More than 7 | Ref |  | Ref |  |
| **Sex** |  |  |  |  |
| Male | Ref |  |  |  |
| Female | 1.15 | 0.87 – 1.52 |  |  |
| **Numbers of rooms in the household** | | | | |
| 1-3 | Ref |  | Ref |  |
| 4-6 | 0.74 | 0.35 – 1.58 | 0.58 | 0.25 – 1.31 |
| More than 7 | 0.29 | 0.06 – 1.53 | 0.20^*^ | 0.05 – 0.80 |
| **Education** |  |  |  |  |
| None | 1.07 | 0.74 – 1.55 | 1.02 | 0.59 – 1.76 |
| Primary | 0.42^***^ | 0.29 – 0.62 | 0.54^*^ | 0.31 – 0.94 |
| High | Ref |  | Ref |  |
| **Age of household members** | | | | |
| 0-5 | Ref |  |  |  |
| 5-10 | 0.74 | 0.43 – 1.29 |  |  |
| 10-20 | 0.71 | 0.47 – 1.06 |  |  |
| 20-40 | 1.62^*^ | 1.07 – 2.46 |  |  |
| 40-50 | 3.12^*^ | 1.36 – 7.17 |  |  |
| 50-60 | 7.14^*^ | 1.68 – 30.24 |  |  |
| + 60 | 3.68^*^ | 1.36 – 9.97 |  |  |
| **Age of household head** | | | | |
| < 30 | 1.79 | 0.50 – 6.38 | 1.35 | 0.41 – 4.44 |
| 30 - 40 | 2.38^*^ | 1.12 – 5.03 | 1.88 | 0.87 – 4.04 |
| 40 - 50 | Ref |  | Ref |  |
| 50 - 60 | 1.42 | 0.56 – 3.59 | 1.27 | 0.52 – 3.07 |
| + 60 | 2.23^*^ | 1.05 – 4.72 | 2.46^*^ | 1.05 – 5.76 |
| **Marital status** | | | | |
| Married | 3.58^***^ | 2.30 – 5.58 | 3.56^***^ | 2.06 – 6.14 |
| Never Married | Ref |  | Ref |  |
| Widowed | 2.64 | 0.74 – 9.42 | 2.42 | 0.68 – 8.57 |
| **Presence of children under five** | | | | |
| Yes | 1.20 | 0.68 – 2.12 |  |  |
| No | Ref |  | Ref |  |
| **Pregnancy status** | | | | |
| Yes | Ref |  | Ref |  |
| No | 0.80 | 0.24 – 2.67 |  |  |
